# Genetic diversity of *Potato virus* M (PVM) in the major potato growing region in the Indo-Gangetic plain and characterization of a distinct strain of PVM occurring in India

**DOI:** 10.3389/fmicb.2023.1265653

**Published:** 2023-11-23

**Authors:** Alok Kumar, Akshay Katiyar, A. Abdul Kader Jailani, Ashis Chackraborty, Bikash Mandal

**Affiliations:** ^1^Division of Plant Pathology, Advanced Centre for Plant Virology, Indian Agricultural Research Institute, New Delhi, India; ^2^Department of Plant Pathology, Bidhan Chandra Krishi Viswavidyalaya, Mohanpur, West Bengal, India

**Keywords:** complete genome, enzyme-linked immunosorbent assay, genetic diversity, Indian isolate, *Potato virus* M, reverse transcriptase polymerase chain reaction

## Abstract

*Potato virus* M (PVM) is one of the most prevalent viruses infecting potatoes worldwide, showing a wide range of diversity in their populations; however, the diversity and genome information of PVM occurring in India is hardly known. The present study serologically detected the PVM in 22.8% of leaf samples collected from the potato fields, generated 13 coat protein (CP) genes and one complete genome sequence for the isolates from India, and identified four differential hosts confirming PVM-Del-144 as a distinct strain of PVM occurring in India. The phylogenetic analyses conducted based on the CP gene sequences (14 from India and 176 from other countries) suggested the existence of three evolutionary divergent lineages (PVM-o, PVM-d, and a new divergent group) in the PVM population, where isolates from India belong to only two clusters (PVM-o and PVM-d) within four sub-clusters. High levels of nucleotide diversity (0.124) and genetic distance (0.142) recorded among the isolates from India may be due to the deviation from the neutral evolution and experiencing population expansion in the past. The complete genome of the isolate Del-144 (KJ194171; 8,526 nucleotides) shared 92.2–93.9% nt sequence identity with the population of PVM-o, whereas it shared only 70.2–72.1% identity with PVM-d. In the phylogenetic analyses, Del-144 clustered with the isolates of PVM-o; however, it formed a separate branch away from all other isolates, indicating the diversity of the strain. Overall, this study revealed the diversity of the isolates of PVM from India and reported the first complete genome sequence of a distinct strain of PVM occurring in India.

## Introduction

Potato (*Solanum tuberosum*) is the world's third most important food crop after rice and wheat and is the staple food of 1.3 billion people (Devaux et al., 2021). The planted area of potatoes increased rapidly and reached 17.3 million hectares (h) globally in 2019, producing over 370 million tons of potatoes, of which India is the 2nd largest contributor after China with 14% of world potato production (FAOSTAT, 2021). The total cultivated area under potato production in India was 2,248,000 h that produced over 54 million tons of potatoes with a total productivity of 24.1 t/h (FAOSTAT, 2021). Indo-Gangetic plain (Bihar, Delhi, Haryana, Madhya Pradesh, Punjab, Uttar Pradesh, and West Bengal) is the major potato growing area in India, which contributes >80% of the total potato production in India (Pandey and Kang, 2003). Potato production in India is constrained by several biotic and abiotic factors, of which viral diseases pose serious limitation as it impacts yield, quality, degeneration of tuber seed stock quality, and disposition to susceptibility to other pathogens (Ali et al., 2013; Awasthi and Verma, 2017; Ghorai et al., 2018; Priegnitz et al., 2019; Adolf et al., 2020; Wasilewska-Nascimento et al., 2020).

So far, approximately 57 viruses have been reported to infect potatoes worldwide, of which only 11 viruses, namely, *Alfalfa mosaic virus* (AMV; Alfamovirus), *Potato virus* X (PVX; Potexvirus), *Potato virus* Y (PVY; Potyvirus), *Potato virus* A (PVA; Potyvirus), *Potato virus* M (PVM; Carlavirus), *Potato virus* S (PVS; Carlavirus), *Potato aucuba mosaic virus* (PAMV; Potexvirus), *Potato leaf roll virus* (PLRV; Polerovirus), *Groundnut bud necrosis virus* (GBNV; Tospovirus), *Potato apical leaf curl virus* (PALCV; Begomovirus), and *Tomato leaf curl New Delhi virus* (TLCNDV; Begomovirus), are reported from India (Khurana and Singh, 1988; Garg and Khurana, 1994; Garg et al., 2001, 2003; Usharani et al., 2004; Kaushal et al., 2007; Mandal et al., 2012; Pundhir et al., 2012; Jeevalatha et al., 2013; Kumar et al., 2014; Halabi et al., 2019, 2021). Among all, PVM, a member of the genus *Carlavirus* in the family *Betaflexiviridae*, is considered to be one of the most prevalent and economically important potato viruses infecting potatoes worldwide (Salazar, 1996; Halterman et al., 2012). The virus is naturally transmitted by aphids in a non-persistent manner, and depending on the cultivars used and environmental conditions, it may infect all the potatoes grown in the field (Cavileer et al., 1998; Xu et al., 2010). It can also be transmitted through sap inoculation to the young leaves of the host plants; however, poor cultural practices are considered to be the major reason for the spread of PVM, causing 25–50% yield losses to the crop (Salazar, 1996; Halterman et al., 2012).

The Carlaviruses have a single-stranded positive-sense RNA genome of 8.3–8.7 kb size with a 5′ cap, followed by six open reading frames (ORFs) and a 3′ poly(A) tail (Morozov and Solovyev, 2003). The virions of PVM are flexuous rods of 650x12 nm in size, which contain a positive-sense single-stranded RNA genome of ~8.5 kb in length (Zavriev et al., 1991; Cavileer et al., 1998; Adams and Antoniw, 2004). Typical to the genome organization of Carlaviruses, the PVM genome also contains a cap structure at the 5′ end followed by six ORFs and a poly-A tail at the 3′ end (Zavriev et al., 1991). The ORF-1 encodes a multi-domain protein responsible for RNA replication, which contains three conserved motifs, namely, methyltransferase, helicase, and RNA-dependent RNA polymerase (RdRp) (Koonin et al., 1993; Ju, 2005). The ORF-2, ORF-3, and ORF-4 overlap each other, forming a so-called triple gene block (TGB), which involves membrane binding and cell-to-cell movement of virions (Carrington, 1996; Morozov and Solovyev, 2003). The TGBp1 protein is also known to be involved in the suppression of RNA silencing (Senshu et al., 2011). The ORF-5 encodes the coat protein (CP), whereas ORF-6 encodes a cysteine-rich nucleic acid-binding protein (NABP) having the ability to bind with the single- or double-stranded RNA and DNA and suppress RNA silencing (Gramstat et al., 1990; Zavriev et al., 1991; Senshu et al., 2011). A recent study has also confirmed the involvement of the N-terminal region of NABP in determining the symptoms caused by the carlavirus (Fujita et al., 2018).

It has also been reported that members of Carlavirus such as PVS, PVM, and hop mosaic virus (HpMV) shown remarkable genetic diversity in their population (Cox and Jones, 2010; Poke et al., 2010; Xu et al., 2010; Salari et al., 2011; Tabasinejad et al., 2014; Plchova et al., 2015). The genetic diversity of PVM has been well studied for the isolates of China and Iran that classified the PVM isolates into two clades: PVM-ordinary (PVM-o) and PVM-divergent (PVM-d) (Tabasinejad et al., 2014). Subsequently, a divergent strain of PVM has been reported from Slovakia that formed a separate lineage away from both the clusters reported earlier (Glasa et al., 2019), similar to the clustering for HpMV where isolates were divided into three groups (Poke et al., 2010). All these studies confirmed that the coat protein (CP) region is the best genomic region for studying the genetic diversity of carlaviruses. The knowledge of the population structure is helpful in understanding the changes in geographical range, pathogenicity, epidemiological routes, mechanism of evolution, possibility of emergence of new strains, and ultimately in designing the strategies for controlling viruses (Acosta-Leal et al., 2011).

Despite the wide occurrence of PVM, the host range of the virus is limited to solanaceous species producing mottling, mosaic, crinkling, leaflet deformation, and stunting of shoots (Xu et al., 2010). However, symptoms of PVM in potato are similar to those caused by other potato-infecting viruses, such as PVS, PVX, and PVY, and vary from mild to severe in case of mixed infection (Heitefuss et al., 1993; Stevenson et al., 2001). The PVM is distributed worldwide; however, the infection of this virus is reported to be more prevalent in Eastern Europe, the former Soviet Union than in North America, and Canada (German, 2001; Balagun et al., 2002; Tripathi, 2004). In India, PVM was first recorded during the late 1970s in Shimla hills based on the symptoms and serology (Khurana and Nagaich, 1975, Khurana and Singh, 1980); however, the genomic properties and diversity of PVM occurring in India are still unknown. Therefore, the present study aimed to investigate the prevalence of PVM in the Indo-Gangetic plain of India, which is the major potato-growing region in the country, and study the genetic diversity of the isolates of PVM occurring in this region. We are also reporting the first complete genome sequence, host reactions, and phylogenetic relationship of a distinct isolate of PVM from the Indo-Gangetic region.

## Materials and methods

### Survey and sampling

Surveys and sampling were conducted in 18 districts of seven major potato-producing states in the Indo-Gangetic region: Bihar, Delhi, Haryana, Madhya Pradesh, Punjab, Uttar Pradesh, and West Bengal during three consecutive years, 2012–2014 (Table 1). In each district, only the commercial potato farms were selected for survey and sample collection, and approximately 10–30 composite samples (1 sample = leaves collected from 3 to 5 plants) were randomly sampled from each location except for the experimental farms at IARI, from where comparatively higher number of samples were collected due to their location advantage, and therefore, the total number of samples collected and tested for each location varied and are mentioned in Table 1. Potato leaves in sufficient quantity were collected in polybags, transported in cold chain to the laboratory at Advanced Center of Plant Virology, Indian Agricultural Research Institute, New Delhi, and stored at 4°C in a refrigerator. Sub-samples were immediately processed for a serological assay (ELISA) to confirm the association of the PVM with the samples, and the remaining material was stored in a −80°C freezer in preparation for molecular analysis.

**Table 1:** Detection of *Potato virus* M by direct antigen-coated enzyme-linked immunosorbent assay (DAC-ELISA) in the field samples collected from different locations of northern plain of India.

**State**	**Place**	**Year of collection**	**No. positive/no. tested (%)**	**OD range for positive samples^a^**
West Bengal	Jalpaiguri	2012	7/10 (70)	0.361–0.741
Bihar	Vaishali	2012	9/21 (42.8)	0.211–0.542
Bihar	Patna	2012	9/22 (40.9)	0.429–0.663
Delhi	IARI field-1	2012	10/27 (37.0)	0.316–0.843
Delhi	IARI field-2	2012	5/85 (5.9)	0.231–0.342
Delhi	IARI field-3	2012	5/85 (5.9)	0.355–1.785
Delhi	IARI field-4	2014	19/53 (35.8)	0.383–1.229
Delhi	IARI field-5	2013	17/160 (10.6)	0.309–0.565
Haryana	Sonipat	2013	11/35 (31.4)	0.275–0.476
Panjab	Jalandhar	2013	7/20 (35.0)	0.386–0.532
Uttar Pradesh	Meerut	2012	13/20 (65.0)	0.402–0.618
Uttar Pradesh	Balia	2012	4/22 (18.2)	0.145–0.186
Uttar Pradesh	Gajipur	2012	2/13 (15.4)	0.175–0.197
West Bengal	Kolkata	2013	3/8 (37.5)	0.216–0.356
Uttar Pradesh	Agra	2013	4/25 (16)	0.307–0.355
Uttar Pradesh	Mathura	2013	3/6 (50)	0.328–0.429
Uttar Pradesh	Jaunpur	2013	4/33 (12.1)	0.137–0.217
Uttar Pradesh	Mirjapur	2013	4/15 (16.7)	0.198–0.697
Uttar Pradesh	Hatras	2014	1/21 (4.8)	0.321
Uttar Pradesh	Kannauj	2014	1/11 (9.1)	0.158
Madhya Pradesh	Indore	2014	18/31 (58.0)	0.249–0.532
Madhya Pradesh	Ujjain	2014	16/30 (53.33)	0.208–0.614
	Total		172/753 (22.8)	

a The value mentioned is after subtracting the value of the buffer control.

### Screening for PVM infection by enzyme-linked immunosorbent assay (ELISA)

A total of 753 potato leaf samples collected from 18 districts of 7 major potato-producing states were screened for the association of PVM by direct antigen-coated ELISA (DAC-ELISA) using virus-specific polyclonal antiserum (Bioreba, Switzerland) following the protocol of Clark and Bar-Joseph (1984). In brief, 200 μl of leaf extracts in 1:10 dilution, prepared in coating buffer containing 2% polyvinyl pyrrolidine (PVP, MW 40,000), were added to the well of microtiter plate followed by blocking the free protein binding sites by 5% skimmed milk dissolved in 1× phosphate buffer saline (PBS). The PVM antiserum diluted in PBS-TPO (PBS with 0.05% Tween 20, 2% PVP, and 0.2% ovalbumin) was added to each well and incubated at 37°C for 1 h followed by incubation with goat anti-rabbit IgG-alkaline phosphatase conjugate (Sigma Aldrich, St. Louis, USA) (1:3,000) at 37°C for 1 h. The results were analyzed by the development of a yellow color following a reaction with freshly prepared substrate solution (0.5 mg/ml) of p-nitrophenyl phosphate (PNPP) prepared in diethanolamine buffer, pH 9.8. The plates were washed with PBST (PBS with 0.05% Tween 20), and washing was carried out after each step. The absorbances of samples were recorded at 405 nm using an ELISA reader (ELX 800 Universal Microplate Reader from BioTek Instruments Inc., Winooski, VT, USA) 1 h after adding the substrate solution to the microtiter plate. The absorbance values double or more of the value of healthy control were considered positive for PVM infection.

### Reverse transcriptase polymerase chain reaction (RT-PCR)

Total genomic RNA was extracted using RNeasy Plant Mini Kit (Qiagen, Chatsworth, CA) following the manufacturer's protocol from the potato leaf samples and showed a positive reaction in DAC-ELISA. The RT-PCRs were conducted in a two-step process. The first strand cDNA was synthesized in a 20 μl reaction mixture containing 4 μl of 5x First-Strand buffer, 1 μl of 10 mM of dNTP mix, 1 μl of 20 mM DTT, 1 μl of 10 μM of reverse primer, 1 μl of (100 units/μl) SMARTScribe^TM^ III reverse transcriptase enzyme (Clontech, USA), and 2 μl of RNA template (400–500 ng), and the final volume was adjusted by adding nuclease-free water. The mixture was subjected to 42°C for 90 min followed by inactivation at 70°C for 15 min using a thermal cycler (Biometra *T* Personal). PCR was conducted in a 50 μl reaction mixture containing 1 μl of cDNA, 5 μl of 10x Ex *Taq* PCR buffer, 4 μl of 2.5 mM dNTPs, 2 μl of 10 μM of each primer, 0.25 μl (1.25 U) of Ex *Taq* DNA polymerase (Takara, Japan), and nuclease-free water to make up the volume. The mixture was subsequently subjected to 35 PCR cycles, each consisting of denaturation at 98°C for 10 s, primer annealing at 52–60°C for 45 s, and extension at 72°C for 1 min/kb with the final extension at 72°C for 10 min.

### Cloning, sequencing, sequence analyses, and genetic distance

The CP gene or the 3′ genome [CP to 3′ untranslated region (UTR)] of PVM was amplified from the ELISA-positive samples collected from the different districts of northern India using the primer pairs BM455F and BM587R or BM455F and BM589R, respectively, for studying the genetic diversity of PVM isolates occurring in India. RT-PCR for the targeted region was conducted as mentioned above. The PCR products were resolved in agarose gel, purified using PCR and gel purification kit (Macherey-Nagel, Germany), cloned in a T&A cloning vector (Real Biotech Corporation, Banqiao, Taiwan) using the manufacturer's protocol, and sequenced from both the directions using the commercial facility at Chromous Biotech (Bengaluru, India). Similarly, the complete genome of one of the PVM isolates (PVM-Del-144) was amplified in five overlapping fragments using five pairs of overlapping primers designed based on the sequences available in the NCBI database (Table 2). The amplified products were resolved in agarose gel and cloned in a T&A cloning vector (Real Biotech Corporation, Banqiao, Taiwan) (Supplementary Figure 1), and two clones of each amplified product were sequenced from both directions. The vector sequences from each clone were removed, analyzed by a basic local alignment search tool (BLAST) (http://www.ncbi.nlm.nih.gov/blast), and aligned with the sequences of other PVM isolates available in the NCBI database using the multiple sequence alignment program ClustalW in BioEdit v.7.2.5 (Hall, 1999).

**Table 2:** List of primers used for the amplification of complete genome of *Potato virus* M isolate (PVM-Del-144) from India.

**Primer name**	**Primer sequence (5^′^- 3^′^)**	**Primer location**	**Annealing temperature (°C)**	**Amplicon length (bp)**
BM588F	taaacaaacatacaatatctggacttacactacaatatactacca	1	60	3,453
BM625R	gcgagcattcctgcgcgattcc	3,453		
BM624F	cggacggcgcaaagacccca	3,310	57	1,521
BM664R	gtcactagctctatgcctggggtatatgg	4,831		
BM665F	agagaggtgcgcatgggagatatg	4,700	58	1,600
BM666R	ggggtcaccgaataatgcaaatacc	6,300		
BM674F	accaaccaaggttgagcg	6,200	52	1,443
BM456R	gcacgtgctcagtcggc	7,643		
BM455F	atctgaaatagtgagtatggg	7,225	56	1,308
BM589R	tggctaaaaatagttaaaaaccaagttttatttatagtagcacac	8,533		

The complete genome of PVM-Del-144 was obtained by joining the sequences of five overlapping fragments using the BioEdit sequence alignment editor (Hall, 1999). The coding sequence of the assembled genome was determined by ORF Finder available at the NCBI website (http://www.ncbi.nlm.nih.gov/gorf/gorf.html). For the genetic diversity study, only the nucleotide (nt) sequences of the CP gene were used for comparison, and pairwise identities were calculated using the Sequence Demarcation Tool (SDT v.1.2) (Muhire et al., 2014). The nucleotide (nt) and deduced amino acid (aa) sequences of the complete genome and each ORF of PVM-Del-144 were compared with the sequences from GenBank isolates, and pairwise identities were calculated using the BioEdit sequence alignment editor (Hall, 1999). The phylogenetic and molecular evolutionary analyses were conducted based on the nt sequences of the CP gene and the full-length genome of PVM, generated in this study and available in the GenBank database, using the maximum-likelihood method of the software MEGA, version 11 (Tamura et al., 2021) with 500 and 1,000 bootstrap values, respectively. The intra- and intergroup genetic distances among PVM isolates were also calculated using MEGA version 11 (Tamura et al., 2021).

### Genetic diversity, population genetics, selection pressure, and neutrality tests

The genetic diversity and population genetics parameters, average nucleotide diversity (π), haplotype diversity (Hd), number of polymorphic or segregating sites (S), the statistic estimate of population mutation based on the number of segregating sites (θ-w), total number of mutations (Eta), the average number of nucleotide differences between sequences (k), and the statistic estimate of population mutation based on the total number of mutations (θ-Eta) within and between populations were calculated using CP gene sequences in DnaSP v6.12.03 (Rozas et al., 2017). The genetic differentiation (*F*__*ST*__, *K*_*ST*_*, Z*, and *S*_*nn*_*)* and level of gene flow (N_m_) between the major identified populations were also measured by DnaSP v6.12.03 (Rozas et al., 2017).

The possible occurrences of selection pressure were estimated by four codon-based approaches, namely, fixed effects likelihood (FEL), single-likelihood ancestor counting (SLAC), fast unconstrained Bayesian approximation (FUBER), and mixed effects model of evolution (MEME), using the Datamonkey software at http://www.datamonkey.org (Pond and Frost, 2005; Kosakovsky-Pond et al., 2011; Murrell et al., 2012; Weaver et al., 2018). These approaches use the dN/dS ratio for determining the selection pressure, where dN represents the average number of non-synonymous substitutions per non-synonymous site and dS represents the average number of synonymous substitutions per synonymous site. If the value of dN/dS < 1, it is considered as purifying or negative selection, dN/dS = 1 represents neutral selection, whereas dN/dS > 1 represents diversification or positive selection pressure.

Three determinants (Tajima's *D*, Fu and Li's *F*, and Fu and Li's *D*) were used to determine the deviation of PVM populations from neutrality assuming all mutations are selectively neutral with zero recombination (Ramírez-Soriano et al., 2008) and were calculated by DnaSP v6.12.03 (Rozas et al., 2017). Tajima's D test identifies evolutionary events, such as population expansion, bottleneck effects, and natural selection, by comparing the number of segregating sites and the average number of nucleotide differences (Tajima, 1989). A negative value for Tajima's *D* statistic indicates superfluous low-frequency polymorphism triggered by background selection, genetic hitchhiking, or population expansions (Alabi et al., 2011), whereas positive statistic values suggest minimal levels of low- and high-frequency polymorphisms, indicating a reduction in population size and/or balancing selection. Fu and Li's D and Fu and Li's F values are sensitive to population expansion and are usually negative when the population is expanded (Fu and Li, 1993).

### Host biology of an Indian isolate of PVM (PVM Del-144)

The host reactions of an isolate of PVM (PVM-Del-144) collected from the experimental field of the Indian Agricultural Research Institute (IARI), New Delhi, in January 2013 were determined by the sap inoculation of the virus isolate to the young seedlings of various plant species following the protocol of Kumar et al. (2022). In brief, the inoculum of the virus was prepared by grinding the infected leaf samples in 1% (w/v) 0.1 M phosphate buffer pH 7.2 and applied on the leaves of the test plants pre-dusted with Carborundum 320 grit. Initially, the isolate was mechanically transmitted to the indicator host, *Phaseolus vulgaris* (Red Kidney bean), where it produces necrotic lesions on inoculated leaves. The local lesion leaves were further used to study the host biology of PVM-Del-144 by sap inoculation to nine plant species listed in Table 3 at 3–5 leaf stage in the greenhouse at a temperature ranging from 20 to 25°C. The inoculation experiment was conducted in three replications, and the inoculated plants were regularly observed for symptom expression. The symptom expression was recorded, and the association of the virus with the inoculated plants was confirmed by reverse transcriptase PCR (RT-PCR) using CP-specific primers to PVM.

**Table 3:** Host reactions of *Potato virus* M isolate from India (PVM-Del-144) and its comparison with isolates from other countries.

**Host plants**	**Symptoms**		**References**
	**PVM-Del-144**	**Other isolates**	
*Solanum tuberosum*	Abaxial leaf rolling, necrosis, and stunting of plant	Mottle, mosaic, crinkling and rolling of leaves, stunting of shoots	Xu et al., 2010
*Phaseolus vulgaris*	Necrotic spots	Local necrotic lesion on primary leaves	Bagnall et al., 1956, 1959; Kowalska and Was, 1976; Edwardson and Christie, 1997
*Datura stramonium*	Local lesion with no systemic symptoms	Local chlorotic spotting and systemic chlorotic mottling and rugosity	Bagnall et al., 1956, 1959; Kowalska and Was, 1976; Edwardson and Christie, 1997
*Nicotiana benthamiana*	Symptomless	Symptomless	Flatken et al., 2008
*Solanum lycopersicum*	Yellowing of leaves	Symptomless	Flatken et al., 2008
*Gomphrena globosa*	No infection	Local cholrotic rings or necrotic spots	Hiruki, 1970
*Chenopodium quinoa*	No infection	Chlorotic local lesions	Xu et al., 2010
*N. glutinosa*	No infection	No infection	Bagnall et al., 1956, 1959; Kowalska and Was, 1976; Edwardson and Christie, 1997
*N. tabacum* cv. Xanthi	No infection	No infection	Bagnall et al., 1956, 1959; Kowalska and Was, 1976; Edwardson and Christie, 1997

## Results

### Field observations and screening for PVM infection

The potato plants in the entire surveyed fields in all the selected districts of northern India showed the symptoms of mosaic, mottling, crinkling, leaf rolling, leaflet deformation, and sometimes stunting. All of 753 samples collected from the fields were screened for PVM infection of which 172 samples tested positive in DAC-ELISA showing the overall 22.8% incidence of PVM in northern India (Table 1). The incidence of PVM in the surveyed districts ranged from 4.8% to 70.0% of which approximately half of them (Jalpaiguri, Vaishali, Patna, Sonipat, Jalandhar, Meerut, Kolkata, Mathura, Indore, Ujjain, and few fields at IARI, New Delhi) showed >30% PVM incidence in the samples collected from the fields.

### Coat protein sequences and comparison

The CP gene sequences of PVM for the isolates from different locations in northern India were obtained from nine clones of the CP gene and four clones of the 3′ genome generated in this study. All the sequences were submitted to the NCBI database whose accession nos. and other details are mentioned in Table 4. All these CP sequences together with the CP sequences of Del-144 were compared among themselves and with 176 CP sequences of PVM reported from other countries and available in the NCBI database. Similar to the majority of the PVM isolates reported worldwide, the CP gene of all the PVM isolates from India was identical (915 bp) in length and shared 74.5–100% nt identity with each other. Based on the nt sequence identity they shared with each other, PVM isolates of India were categorized into two clades. The clade1 (PVM-o) consists of 10 isolates (Del-144, Mat-12, Kan-16, Agf-5, Del-147, Del-134, Del-123, Mir-12, M34, Gaj-13), shared 92.4–100% identity among themselves whereas, and clade2 (PVM-d) contains 4 isolates (Del-133, Jau-13, Hat-12, Bal-21), shared 94.3–98.1% identity among themselves, but only 74.5–76.8% identity with the isolates from clade1 (Supplementary Figure 2).

**Table 4:** List of the *Potato virus* M isolates of India, their sampling location, fragment size, and accession numbers used in this study for comparison.

**S. No**.	**Isolate name**	**Location of sampling**	**Sequence length (bp)**	**NCBI accession no**.
1	Agf-5	Agra	924	KJ919964
2	Bal-21	Balia	924	KJ462133
3	M34	Delhi	924	KF471070
4	Del-147	Delhi	924	KJ462137
5	Del-134	Delhi	924	KJ462136
6	Del-123	Delhi	924	KJ462135
7	Del-133	Delhi	1,309	KJ569696
8	Gaj-13	Gajipur	1,309	KJ473992
9	Hat-12	Hatrus	924	KJ919966
10	Jau-13	Jaunpur	1,300	KJ473993
11	Kan-16	Kannauj	924	KJ919965
12	Mat-12	Mathura	1,309	KJ569697
13	Mir-12	Mirjapur	924	KJ462134

The comparison of PVM-CP sequences of the isolates from India with the isolates reported worldwide showed that the isolates of India from PVM-o clade shared a maximum of 96.0% identity with the isolates from China, 97.8% with isolates from Iran and Russia, 97.5% with isolates from European countries, and 97.4% with lone isolate from Tanzania, whereas it shared only a maximum of 75.7%, 76.1%, and 74.7% identities with the isolates from the United State of America, Bangladesh, and with lone isolate of Japan, respectively. The comparison of the isolates of clade2 (PVM-d) from India with the isolates of the same clade reported worldwide divided the clade2 into three sub-clades, sharing >91%, 79.8–81.6%, and <76.2% identity, respectively. However, the isolates only from above mentioned a maximum of any two sub-clades were found to present in one region/country. For example, Iran and European countries have isolates from sub-clade-2b and sub-clade-2c shared a maximum of 80.6% and 75.1%, and 80.7% and 75.5% identity, respectively, whereas Bangladesh and Canada have isolates from sub-clade-2a and sub-clade-2c shared a maximum of 95% and 82%, and 93.1% and 76.2% identity, respectively. The lone isolate of PVM-d from China and Japan shared 94.6% and 94.7% identity, respectively. However, all the PVM isolates shared <53% identity when compared with the CP sequences of *Potato virus S* (PVS), which is the closest member of PVM under the genus Carlavirus. It has also been observed that the isolate Gaj-13 from India technically belongs to PVM-o; however, it shared the least identity with the isolates from India and worldwide.

**Figure 1:**
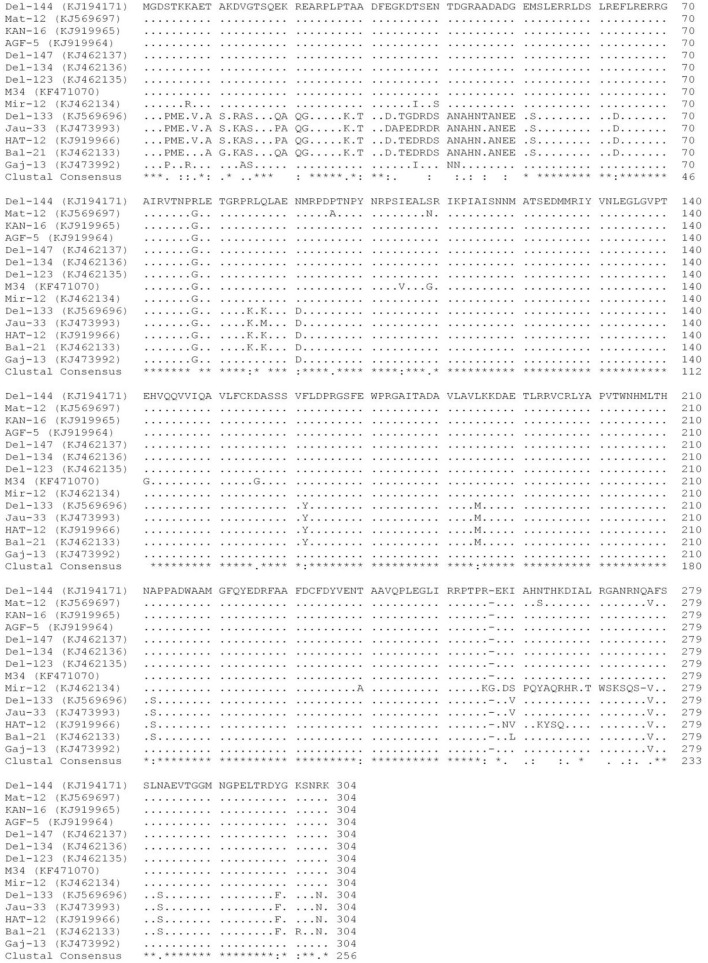
Multiple alignments of the amino acid sequences of the coat protein of 14 isolates of *Potato virus* M reported from India. Dots (.) represent identical sequences; dashes (-) represent missing sequences; *represents the identity among all the isolates. The place of the origin of each isolate is listed in Table 4.

A pairwise comparison of the aa sequences of the CP gene of PVM isolates from India revealed that most of the variations were found at the N-terminal region, whereas C-terminal was likely to be conserved except for the isolate Mir-12. This comparison confirms that nine isolates of clade1 (Del-144, Mat-12, Kan-16, Agf-5, Del-147, Del-134, Del-123, Mir-12, and M34) are similar to each other and are very much dis-similar from the four isolates (Del-133, Jau-13, Hat-12, and Bal-21) of clade2, whereas the isolate Gaj-13 showed significant dissimilarities from the isolates of both the clades (Figure 1).

### Phylogenetic analyses and genetic distance

**Figure 2:**
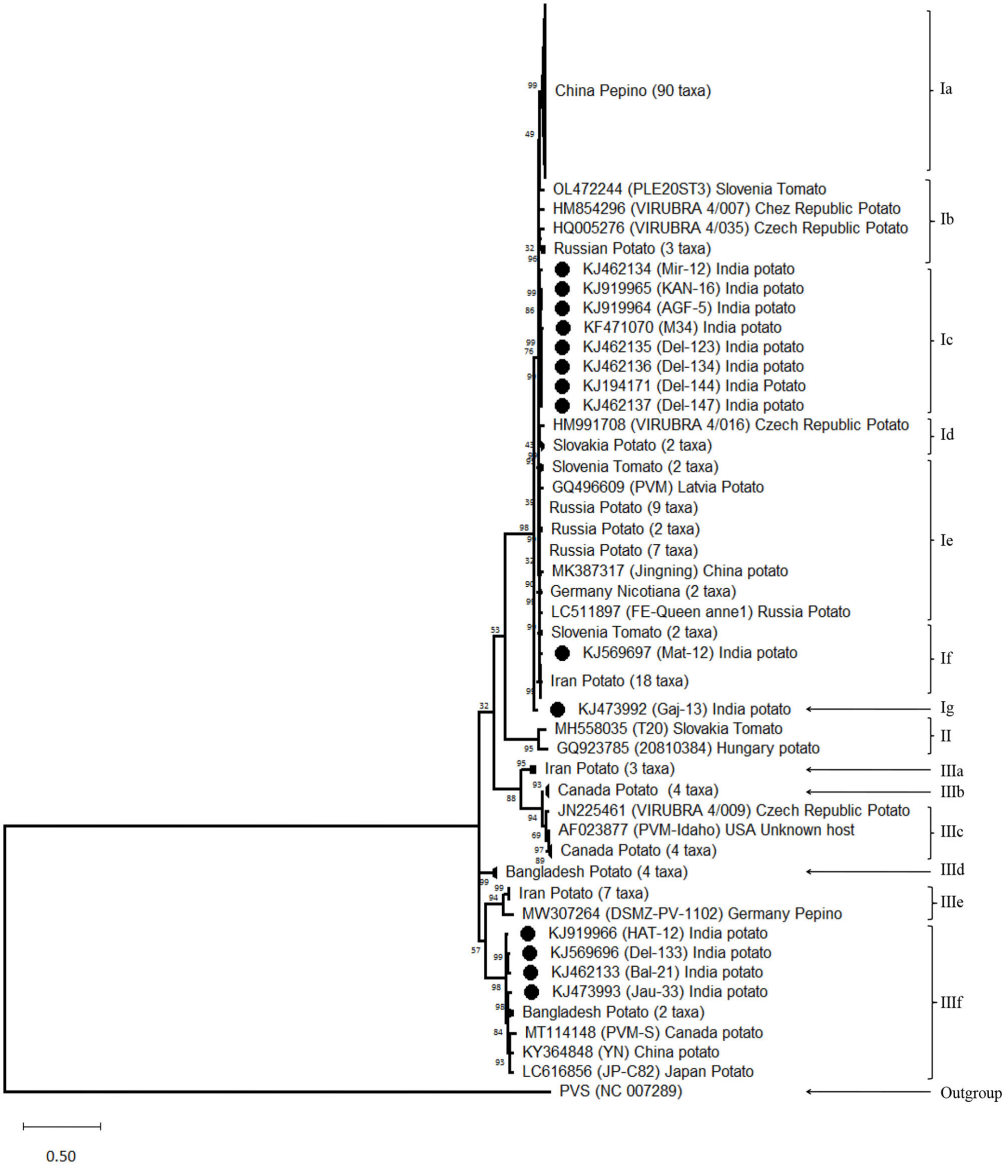
Phylogenetic tree derived from nucleotide sequences of the coat protein gene of *Potato virus* M isolates reported worldwide together with a representative sample of closely related species PVS as an out-group. The tree was constructed by the maximum likelihood method with 1,000 bootstrap replicates using the software MEGA version 11. Bootstrap values are shown at the internodes. Isolates showing higher similarity in a sub-group collapsed for better visibility.

The phylogenetic analyses conducted using the nt sequences of the CP gene of PVM (14 from India and 176 from other countries) together with the most closely related species PVS indicated the diversity among the isolates of PVM and broadly segregated them into three groups (Figure 2). Out of 190 isolates reported worldwide, 154 belong to group-I (PVM-o), 2 belong to group-II (a new divergent group), and 34 belong to group-III (PVM-d). In this analysis, out of 14 isolates from India, 10 isolates belong to PVM-o and the remaining 4 belong to PVM-d (Figure 2). However, further analysis of the phylogenetic tree divided group-I and group-II into 7- and 6-sub-groups, respectively, which are designated by the alphabetical letters, a to g. It has also been observed that isolates originating from one country usually formed a separate cluster within the group and sub-groups with few exceptions. For example, out of 14 isolates from India, 8 isolates clustered together in sub-group-Ic and 4 clustered in sub-group-IIIf, whereas an isolate, Mat-12, clustered with the isolates from Slovenia and Iran in sub-group-If, and a comparatively divergent isolate, Gaj-13, formed a new sub-group-Ig (Figure 2). The similar pattern was observed for the isolates from China and other country/region. All the isolates of China clustered together to form sub-group-Ia, whereas a single isolate, YN, clustered in sub-group-IIIf with the isolates from India, Bangladesh, Canada, and Japan. However, no such sub-grouping was observed in a newly formed divergent group (group-II) that momentarily contains only two isolates, T20 from Slovakia and 20810384 from Hungary. In this phylogenetic analysis, as expected, the most closely related species, PVS, formed a completely separate lineage out of all the phylogenetic groups (Figure 2).

The average evolutionary divergence among the entire population was 0.128 ± 0.007, while it was quite different for the population divided based on different criteria. For example, for the population divided based on the phylogenetic group, the genetic distance within Group I (PVM-o) was 0.050 ± 0.004, whereas it was two (0.094 ± 0.012) and four (0.215 ± 0.013) times higher for Group II (New) and Group III (PVM-d), respectively (Table 5). Within Group I, the minimum genetic distance was recorded for subgroup IF (0.009 ± 0.001), whereas the highest was recorded for subgroup IB (0.049 ± 0.005), which is similar to the overall genetic distance within the population of Group I. Within Group III, the genetic distances within the isolates of each subgroup were found much lesser (0.019–0.094) than the overall genetic distances within the entire population (Table 5). Similarly, the genetic distances calculated for the population categorized based on the host plants they infect were found to be 0.171 ± 0.010 for the population infecting potato plants and 0.034 ± 0.003 for the population infecting pepinoes (Table 6). While calculating the divergence within the PVM population categorized based on the country from where they were reported, the highest distance (0.144 ± 0.010) was found for the population from Iran, followed by India (0.142 ± 0.010), Europe (0.136 ± 0.008), Bangladesh (0.125 ± 0.010), America (0.098 ± 0.007), China (0.040 ± 0.003), and Russia (0.025 ± 0.003) (Table 6).

**Table 5:** Genetic distances within and between phylogenetic groups, and sub-groups of *Potato virus* M isolates based on the CP nucleotide sequences.

**Distances within and between groups**														
**Phylo-groups (No. of isolates)**	**Within groups**	**Between groups (PVM-o) Gr.I**	**(New) Gr.II**											
**(PVM-o) Group I (154)**	0.050 ± 0.004													
**(New) Group II (2)**	0.094 ± 0.012	0.290 ± 0.020												
**(PVM-d) Group III (34)**	0.215 ± 0.013	0.283 ± 0.017	0.293 ± 0.017											
Average evolutionary divergence among entire populations				0.128 ± 0.007										
**Distances within and between sub-groups**														
**Phylo sub-groups**	**Within sub-groups**	**Between sub-groups**												
		**IA**	**IB**	**IC**	**ID**	**IE**	**IF**	**IG**	**II (New)**	**IIIA**	**IIIB**	**IIIC**	**IIID**	**IIIE**
Sub-group IA (90)	0.034 ± 0.003													
Sub-group IB (6)	0.049 ± 0.005	0.072 ± 0.006												
Sub-group IC (8)	0.012 ± 0.002	0.065 ± 0.007	0.054 ± 0.006											
Sub-group ID (3)	0.034 ± 0.005	0.067 ± 0.007	0.052 ± 0.006	0.048 ± 0.006										
Sub-group IE (25)	0.021 ± 0.002	0.065 ± 0.007	0.046 ± 0.005	0.039 ± 0.006	0.045 ± 0.006									
Sub-group IF (21)	0.009 ± 0.001	0.067 ± 0.007	0.051 ± 0.006	0.041 ± 0.006	0.048 ± 0.006	0.051 ± 0.004								
Sub-group IG (1)	NA	0.091 ± 0.010	0.073 ± 0.009	0.070 ± 0.009	0.069 ± 0.009	0.073 ± 0.009	0.067 ± 0.009							
Group II (New) (2)	0.094 ± 0.011	0.292 ± 0.020	0.281 ± 0.020	0.290 ± 0.020	0.280 ± 0.020	0.281 ± 0.020	0.288 ± 0.020	0.266 ± 0.019						
Sub-group IIIA (3)	0.028 ± 0.004	0.267 ± 0.019	0.254 ± 0.019	0.258 ± 0.020	0.257 ± 0.019	0.249 ± 0.019	0.255 ± 0.020	0.244 ± 0.019	0.275 ± 0.020					
Sub-group IIIB (4)	0.015 ± 0.003	0.311 ± 0.022	0.296 ± 0.020	0.300 ± 0.021	0.291 ± 0.021	0.291 ± 0.020	0.291 ± 0.021	0.275 ± 0.020	0.299 ± 0.021	0.196 ± 0.017				
Sub-group IIIC (6)	0.019 ± 0.002	0.294 ± 0.020	0.291 ± 0.020	0.289 ± 0.020	0.285 ± 0.019	0.282 ± 0.020	0.283 ± 0.020	0.276 ± 0.020	0.290 ± 0.021	0.206 ± 0.017	0.081 ± 0.009			
Sub-group IIID (4)	0.021 ± 0.004	0.289 ± 0.021	0.275 ± 0.020	0.283 ± 0.020	0.270 ± 0.020	0.274 ± 0.019	0.278 ± 0.020	0.264 ± 0.019	0.274 ± 0.019	0.263 ± 0.020	0.306 ± 0.021	0.301 ± 0.020		
Sub-group IIIE (8)	0.025 ± 0.002	0.271 ± 0.020	0.259 ± 0.018	0.265 ± 0.019	0.259 ± 0.019	0.257 ± 0.018	0.262 ± 0.019	0.272 ± 0.020	0.299 ± 0.020	0.267 ± 0.020	0.288 ± 0.021	0.269 ± 0.020	0.222 ± 0.018	
Sub-group IIIF (9)	0.056 ± 0.005	0.296 ± 0.020	0.285 ± 0.020	0.295 ± 0.021	0.281 ± 0.020	0.285 ± 0.020	0.288 ± 0.020	0.284 ± 0.020	0.304 ± 0.020	0.255 ± 0.021	0.289 ± 0.020	0.289 ± 0.020	0.214 ± 0.017	0.230 ± 0.017

**Table 6:** Genetic distances within and between host-based and country-based groups of *Potato virus* M isolates calculated using CP nucleotide sequences.

**Distances within and between groups/sub-groups**		
**Grouping based on the host plants they infect**		
**Host-based groups (no. of isolates)**	**Within groups**	**Between groups**
**Potato group**
Potato-group (97)	0.171 ± 0.010	
Pepino-group (91)	0.034 ± 0.003	0.145 ± 0.009
**Grouping based on the isolates from different**		
**Countries/Continent**		
**Name of country/Continent (no. of isolates)**	**Within groups**	**Between isolates from India and other Countries**
India (14)	0.142 ± 0.010
Europe (19)	0.136 ± 0.008	0.149 ± 0.009
China (90)	0.040 ± 0.003	0.129 ± 0.009
Iran (28)	0.144 ± 0.010	0.164 ± 0.010
America (10)	0.098 ± 0.007	0.287 ± 0.018
Bangladesh (6)	0.125 ± 0.010	0.249 ± 0.016
Russia (21)	0.025 ± 0.003	0.109 ± 0.007
Japan (1)	NA	0.227 ± 0.017
Tanzania (1)	NA	0.106 ± 0.008
Out-group (PVS)	NA	0.683 ± 0.040

The genetic distances calculated by MEGA version 11.

The estimation of inter-group genetic distance indicated that the newly assigned phylogroup (Group II) is almost equally distant from the previously reported two other phylogroups (~0.290 ± 0.020); however, further analyzing the distance from each subgroup indicated that subgroup-IIIF showed maximum distance (0.304 ± 0.020) with the isolates of Group II. It has also been observed that inter-subgroup distance within the subgroups under Group III is much higher than that of Group I, which showed higher diversity within the population of Group III (Table 5). Similarly, the newly formed phylogroup (Group II) showed higher distances from the population infecting potato (0.286 ± 0.010) and pepino (0.293 ± 0.022) plants as compared to the distance (0.145 ± 0.020) between potato and pepino groups (Table 6). While comparing the distance between PVM population from India with the population from other country/region, the highest distance was found from the isolates from America (0.287 ± 0.018), Bangladesh (0.249 ± 0.016), and Japan (0.227 ± 0.017), the least was with the lone isolate from Tanzania (0.106 ± 0.008) and with the isolates from Russia (0.109 ± 0.007), and the isolates of China, Europe, and Iran showed intermediate distances from the isolates of India. The out-group containing PVS was most distant with the PVM population from India (Table 6).

### Analyses of genetic diversity, population genetics, genetic differentiation, and gene flow

In the study conducted for estimating the genetic diversity, population genetics, genetic differentiation, and gene flow within and between populations demarcated based on phylogeny, host plants, and country of origin of the isolates, the highest (1.000) haplotype diversity (Hd) was found within the newly formed phylogroup (Group III) containing only two isolates or within the isolates from the United State of America, whereas the least diversity (0.910) was found for the population from Iran (Table 7). However, among the two major PVM populations (PVM-o and PVM-d), the higher haplotype diversity was found within PVM-o (Hd = 0.996) when compared with PVM-d (Hd = 0.988). Although the isolate from India is distributed in both the phylogenetic population, the haplotype diversity only within the Indian population was 0.989, which ranked fourth after USA (1.000), China (0.996), and Europe (0.994). However, the haplotype diversity for the population divided based on the host plants does not show much difference (Table 7). Contrastingly, the number of segregating sites (S = 352), mutations (Eta = 432), and nucleotide diversity (π = 0.04761) for PVM-o was found less than the other major population (PVM-d), where these values were 382, 575, and 0.18561, respectively, indicating higher diversity in PVM-d, although the values of S (456) and Eta (763) were found higher when calculated for the entire population, suggesting many of the mutations and segregating sites are different for each population (Table 7). Interestingly, the nucleotide diversity estimated for the entire population (0.10984) was found to be more than double the diversity of PVM-o (0.04761) but almost half (0.18561) of the PVM-d, which might be because of the inclusion of the third population (Group III) (Table 7). Based on the values of the abovementioned determinants (S, Eta, and π), higher diversity has been observed in the population infecting potato plants compared to the isolates infecting pepinoes (Table 7). The estimation of diversity in the population divided based on the country of their origin indicated that the PVM population from India ranked 4th based on the value of S (284) and Eta (329) after Europe, China, and Iran, whereas it ranked 2nd after Iran based on the value of nucleotide diversity (π = 0.12398). However, the least diversity (S = 115; Eta = 123; π = 0.0256) was recorded for the population from Russia (Table 7).

**Table 7:** Genetic variability determinants and Neutrality tests performed by DnaSP v.6.12 for individual group, and the entire *Potato virus* M population using CP nucleotide sequences.

**Populations (No. of isolates)**	**H**	**Hd**	**N**	**S**	**Eta**	**π**	**k**	**θ-Eta**	Tajima's D^*^	Fu and Li's D^**^	Fu and Li's F^**^
**Based on phylogeny**											
(PVM-o) Group I (154)	131	0.996	882	352	432	0.04761	41.99066	0.08729	−1.48488	−3.41997	−2.98515
(New) Group II (2)	2	1	912	80	80	0.08772	80	0.08772	–	0	0
(PVM-d) Group III (34)	30	0.988	911	382	575	0.18561	169.089	0.15437	0.77567	0.98837	1.0867
**Based on hosts**											
Potato group (97)	81	0.991	875	415	689	0.14068	123.09794	0.15299	−0.27433	0.28236	0.04496
Pepino group (91)	81	0.996	912	349	427	0.03976	36.2591	0.09212	−1.94262	−4.47464	−4.05647
**Based on country/region**											
India (14)	13	0.989	913	284	329	0.12398	113.1978	0.11331	0.42533	0.40804	0.4747
Europe (19)	18	0.994	877	346	506	0.11561	101.39181	0.16508	−1.26522	−0.53507	−0.87911
China (90)	80	0.996	915	366	450	0.04101	37.52609	0.09697	−1.97522	−4.44015	−4.0519
Iran (28)	18	0.91	915	319	382	0.12643	115.68519	0.10728	0.70251	1.39568	1.37808
America (10)	10	1	915	263	288	0.08767	80.22222	0.11126	−1.05998	−1.38884	−1.47714
Bangladesh (6)	5	0.933	915	209	221	0.11417	104.46667	0.10578	0.51522	0.82267	0.8328
Russia (21)	18	0.986	915	115	123	0.0256	23.42381	0.03736	−1.28624	−0.90971	−1.19874
**All (190)**	**163**	**0.99671**	**875**	**456**	**763**	**0.10984**	**96.10805**	**0.14979**	**−0.86106**	**0.09078**	**−0.4538**

H: number of haplotype.

Hd: haplotype diversity.

N: number of nucleotide sites.

S: total number of variable or segregation sites.

Eta: total number of mutations.

π: nucleotide diversity.

k: average number of nucleotide differences between sequences.

θ-Eta: Waterson's estimate of population mutation rate based on the total number of mutations.

Tajima's D^*^: determinant of neutrality test. Calculated by DnaSP v.6.12 using the p-value 0.10 > P > 0.05.

Fu and Li's D^**^ and Fu and Li's F^**^: Determinant of neutrality test. Calculated by DnaSP v.6.12 using the p-value P > 0.10.

The genetic differentiation analyses performed for the major populations divided based on the phylogenetic group, host plants they infect, and country/region from where they were isolated estimated the values of *K*_*ST*_*, Z, S*_*nn*_, and *F*_*ST*_ significant, indicating statistically significant differences between populations (Table 8). Hence, the null hypothesis that the populations are not genetically differentiated was rejected. The estimated low value of N_m_ for the populations divided based on phylogeny (N_m_ = 0.200) and for the population divided according to the country of the origin for the isolates (N_m_ = 0.260) indicates that the genetic drift is easy to occur and that gene flow is not frequent between the populations, thus leading to remarkable genetic differentiation between the populations; however, the value of the same for the population divided based on the host plants (N_m_ = 0.620) was much higher, indicating the genetic drift is difficult and gene flow is frequent between the population divided based on their host plants (Table 8).

**Table 8:** Statistics determined by DnaSP version 6.12.03 for determining the gene flow and genetic differentiation between major identified populations.

**Population**	K${\;}_{\text{ST}}^{*}$	Z^*^	**S_nn_**	**F_ST_**	**N_m_**
Between phylogenetic groups	0.08674	8.52145	1	0.55918	0.2
Between groups created using host plants they infect	0.0776	8.27682	0.98936	8.27682	0.62
Between groups created based on the country of isolation	0.17163	7.84078	0.93617	0.49021	0.26

Analysis was performed by using CP nucleotide sequences. A total of 456 segregating sites were used in the calculations. The genetic differentiation determinants such as, K${\;}_{\text{ST}}^{*}$ and Z^*^ were calculated based on HBK 1992 whereas, S_nn_ was calculated based on Hudson, 2000 using the p-value ^*^0.01 < P < 0.05; ^**^0.001 < P < 0.01; ^***^P < 0.001. The F statistics and N_m_ value for determining the gene flow was calculated based on Hudson et al. (1992).

### Estimation of selection pressure and neutrality of PVM isolates

The number of negative (purifying) selection pressure sites identified by the approaches, namely, fixed effects likelihood (FEL), single-likelihood ancestor counting (SLAC), and fast unconstrained Bayesian approximation (FUBER) on the online Datamonkey server, was 120, 18, and 147, respectively, whereas not a single positive (diversifying) selection pressure site was identified by these methods for the isolates of PVM from India (Table 9). Although the MEME approach identified seven sites under positive selection pressure (Table 9), the total number of sites under negative selection pressure was much higher, indicating that the CP region of PVM isolates of India is mainly under the pressure of purifying selection. The statistical values of Tajima's *D*, Fu and Li's *D*, and Fu and Li's *F* were significantly either negative or positive for all the populations divided based on different criteria except for the population of phylogenetic Group II (Table 9), indicating that PVM populations deviate from neutral mutation, and populations may not be stable. The positive values of Fu and Li's D and Fu and Li's F for the population of phylogroup III (PVM-d), population infecting potato plants, and populations from India, Iran, and Bangladesh indicated that populations are not fully expanded, and it may expand during the course of the evolutionary period.

**Table 9:** Number of selection pressure sites detected in 14 *Potato virus* M isolates from India.

Sites^*^	**Purifying/negative selection**			**Diversifying/positive selection**			
	**FEL**	**SLAC**	**FUBER**	**FEL**	**SLAC**	**FUBER**	**MEME**
304	120	18	147	0	0	0	7

* Total number of codons used in analyses. Analyses were performed on online datamonkey server.

FEL, fixed effects likelihood; SLAC, single-likelihood ancestor counting; FUBER, fast unconstrained Bayesian approximation; MEME, mixed effects model of evolution.

### Complete genome sequence of PVM-Del-144 and its comparison with other PVM isolates

The genome of PVM-Del-144 was 8526 nt long (Accession no. KJ194171) excluding the 3′ poly (A) tail, which contained six open reading frames (ORFs). The UTRs at 5′ and 3′ termini were 75 and 53 nt long, respectively. The ORF1 started with ATG at position 76 and terminated with UAA at position 5,973 nt, encoding a 223 kDa replication protein. The ORF2 spanned between 6,012 and 6,701 nt, encoding TGB1. The ORF3 (6,679–7,008 nt) overlapped with ORF2 by 22 nt, encoded TGB2 and ORF4 (7,005–7,196 nt) overlapped with the ORF3 by 3 nt, encoded TGB3. These three ORFs together formed so-called TGB proteins of 25.4, 11.9, and 6.7 kDa, respectively. The ORF5 (7,218–8,132 nt) encoded the CP of 33.9 kDa and ORF 6 (8,129–8,473 nt) encoded 12.9 kDa NABP.

The complete genome of PVM-Del-144 was compared with 26 other complete genome sequences of PVM available in the NCBI database reported from other countries (Table 10). The complete genome of PVM available in the NCBI database is 1–62 nt smaller than PVM-Del-144 except for YN and wild Russian isolate, which is 5 and 8 nt, respectively, longer than the present isolate. The comparison of the complete genome sequences of 27 PVM isolates reported worldwide broadly divided them into two clusters. The cluster-I includes the isolates from Iran, Czech Republic, Poland, Iran, Germany, Russia, and Slovenia, and share 92.2–93.9% identity with PVM-Del-144, whereas the isolates from Canada, Bangladesh, and Germany belong to cluster-II and shared 70.2–72.1% identity with the isolate Del-144. However, the isolates of China and Slovakia were distributed in both clusters (Table 10). The comparison of the nt and aa sequences of the coding regions, and nt sequences of 5 UTR also confirms the above clustering with only the exception for the isolate, KOR20ST1 that shared far below the expected identity in NABP, which may be due to incomplete terminal sequences (Accession no. OL472243) (Table 10).

**Table 10:** Percent sequence identity of PVM-Del-144 isolate (KJ194171) with the isolates of *Potato virus* M reported from other countries.

**Isolate Name**	**Accession no**.	**Country**	**Host**	**Genome length**	**Complete genome**	**5^′^UTR**	**3^′^UTR**	**Replicase**	**TGB1**	**TGB2**	**TGB3**	**CP**	**NABP**
								**nt/aa**	**nt/aa**	**nt/aa**	**nt/aa**	**nt/aa**	**nt/aa**
T40	MH558037	Slovakia	Tomato	8,523	93.4	97.3	69	93/94.6	92.8/97.8	95.4/98.1	94.7/98.4	95/97.3	90.7/91.2
T50	MH558036	Slovakia	Tomato	8,523	93.6	97.6	69	93.2/94.9	92.8/98.6	94.8/98.1	93.7/98.4	95.3/97.6	90.7/91.2
PVM-352	JX678982	Iran	Potato	8,523	93.8	99	71.8	93.1/94.2	94.2/96.9	94.8/97.2	94.7/93.6	95.9/99	92.7/92.9
Gansu	JN835299	China	Tomato	8,520	92.8	98	96.2	92.3/93.7	93/96.9	94.8/97.2	94.7/95.2	93.2/98	97.6/96.4
VIRUBRA 4/007	HM854296	Czech-Republic	Potato	8,474	92.2	97.6	39.4	92.1/93.9	92.8/95.6	93.9/97.2	95.8/95.2	93.9/97.6	92.7/92.1
M57	AY311395	Poland	Unknown	8,522	93.6	98.6	98.1	93.1/94.2	94.7/99.1	93/95.4	94.7/95.2	94.7/98.6	96.8/96.4
PVM Uran	AY311394	Iran	Potato	8,522	93.4	98	98.1	93/93.9	93.1/96.9	95.1/97.2	93.2/93.6	94/98	97.3/96.4
DSMZ-PV0273	EU604672	Germany	Nicotiana hesperis	8,523	93.8	99	71.8	93.2/93.9	93.6/96.5	94.8/96.3	94.2/93.6	96.2/99	93.3/92.9
DSMZ-PV-0273	MW582794	Germany	Potato	8,523	93.9	98.3	71.8	93.3/94.3	93.4/96.5	95.1/97.2	93.7/93.6	95.9/98.3	93/92.9
Wild Russian	D14449	Russia	Potato	8,533	93.1	97.6	74.6	92/92.4	94.3/97.8	94.5/97.2	92.7/88.8	95.6/97.6	91/88.5
BER20ST2	OL472246	Slovenia	Tomato	8,507	93.6	98.3	59.7	93.2/94.9	94/97.8	94.5/98.1	93.7/93.6	95/98.3	91.5/92.9
KOR20ST2	OL472245	Slovenia	Tomato	8,516	93.7	99	71.8	93.2/94.6	94/97.8	95.1/99	94.2/93.6	95.6/99	92.1/92.1
PLE20ST3	OL472244	Slovenia	Tomato	8,513	93	98.3	70.4	92.4/94.2	93.7/97.3	95.1/99	95.3/98.4	95/98.3	90.7/91.2
KOR20ST1	OL472243	Slovenia	Tomato	8,464	93.2	99	7.3	93.2/94.6	94/97.8	95.1/99	94.2/93.6	95.6/99	44/5.2
KOR20AT	OL472242	Slovenia	Tomato	8,514	93.6	97.6	70.4	93.1/94.6	94/97.8	95.4/99	94.2/93.6	95/97.6	92.4/92.1
T20	MH558035	Slovakia	Tomato	8,524	72.1	85.1	67.6	71.4/78.1	73/79.4	69/72.4	67.1/68.2	75/85.1	71.3/70.1
YN	KY364848	China	Potato	8,530	71.3	84.5	69.4	70.2/76.3	74.7/81.2	71.2/71.5	57/55.3	74.5/84.5	72.7/72.8
PVM-K	MT114149	Canada	Potato	8,479	70.2	86.8	19.7	69.6/75.9	74.4/80.3	69/77	61.9/69.8	74.3/86.8	69.2/69.2
PVM-S	MT114148	Canada	Potato	8,469	70.7	84.5	18.3	70.3/76.7	74.6/81.2	70.9/71.5	56.5/56.9	73.6/84.5	72.1/73.6
PVM-171	MG356509	Bangladesh	Potato	8,475	71.3	85.8	18.3	70.9/77.6	73.3/82	70.9/72.4	61.4/60.3	74.9/85.8	73.6/73.6
PVM-165	MG356508	Bangladesh	Potato	8,477	71.3	85.8	19.7	70.9/77.5	73.3/82	70.9/72.4	61.4/60.3	74.9/85.8	73.6/73.6
18-4	MF133530	Bangladesh	Potato	8,514	71.4	85.5	70.8	70.5/77.6	73/80.7	70.9/72.4	61.4/60.3	75.5/85.5	73/73.6
18-2	MF133529	Bangladesh	Potato	8,511	71.4	84.2	69.4	70.6/77.2	74.4/80.7	72.4/72.4	54.5/52.3	74.8/84.2	71.5/71.9
16-3	MF133528	Bangladesh	Potato	8,508	71.5	85.5	69.4	70.4/76.9	75.2/81.2	73/73.3	54.5/52.3	74.9/85.5	71.8/72.8
16-1	MF133527	Bangladesh	Potato	8,517	71.5	85.8	69.4	70.5/77.4	72.7/80.3	70.9/73.3	60.9/58.7	75.5/85.8	73.3/73.6
DSMZ-PV-1102	MW307264	Germany	Pepino	8,525	71.1	87.1	69.4	69.7/76.3	73.6/80.7	70.6/73.3	59.8/52.3	76.5/87.1	72.7/71.9
PVS	C_007289	Germany	Potato	8,478	51.8	44	38	50.4/41.1	54.2/51.4	58.8/53.6	43.1/25.7	51.4/44	40.1/34.2

The 5 UTR of PVM was found to be more conserved (>84% identity) than that of 3′ UTR (7.3-98.1% identity). Among the coding region, TGB1 and CP gene was found to be more conserved and shared >92.8% nt and >95.6% aa sequence identity within the isolates of cluster-I and >72.7% nt and >79.4% aa sequence identity within the cluster-II, whereas TGB3 was found to be most diverse and shared a minimum of 88.8% and 52.2% aa sequence identity in cluster-I and cluster-II, respectively.

**Figure 3:**
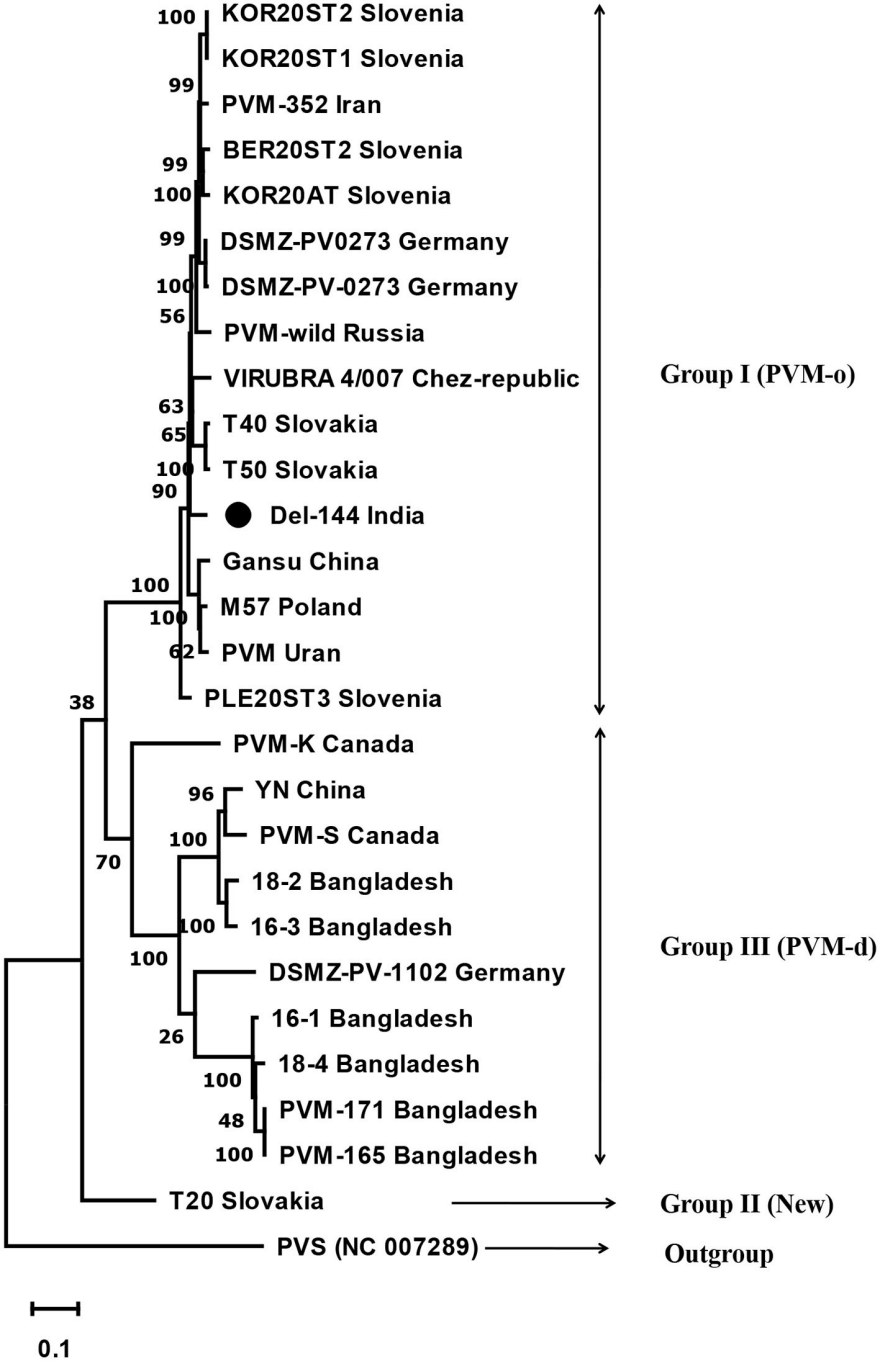
Phylogenetic tree derived from the complete genome sequence of *Potato virus* M isolates reported from India and other contries together with a representative isolate of PVS as an out-group. The tree was constructed based on the maximum likelihood method using MEGA version 11 with a 1000 bootstraps value. The name of the country from where the isolates are reported is mentioned in the tree after the name of the isolate.

The phylogenetic analyses conducted based on the complete genome sequences using the maximum-likelihood method divided the PVM isolates into three groups. Group-I contains 16 isolates including the present isolate, Del-144; however, the present isolate formed a separate branch away from all other isolates in the group. Group-III contains 10 isolates, whereas a lone isolate, T20, formed a new divergent Group-II, which is closely related to the isolates of Group-III (Figure 3).

### Host reactions of PVM-Del-144

**Figure 4:**
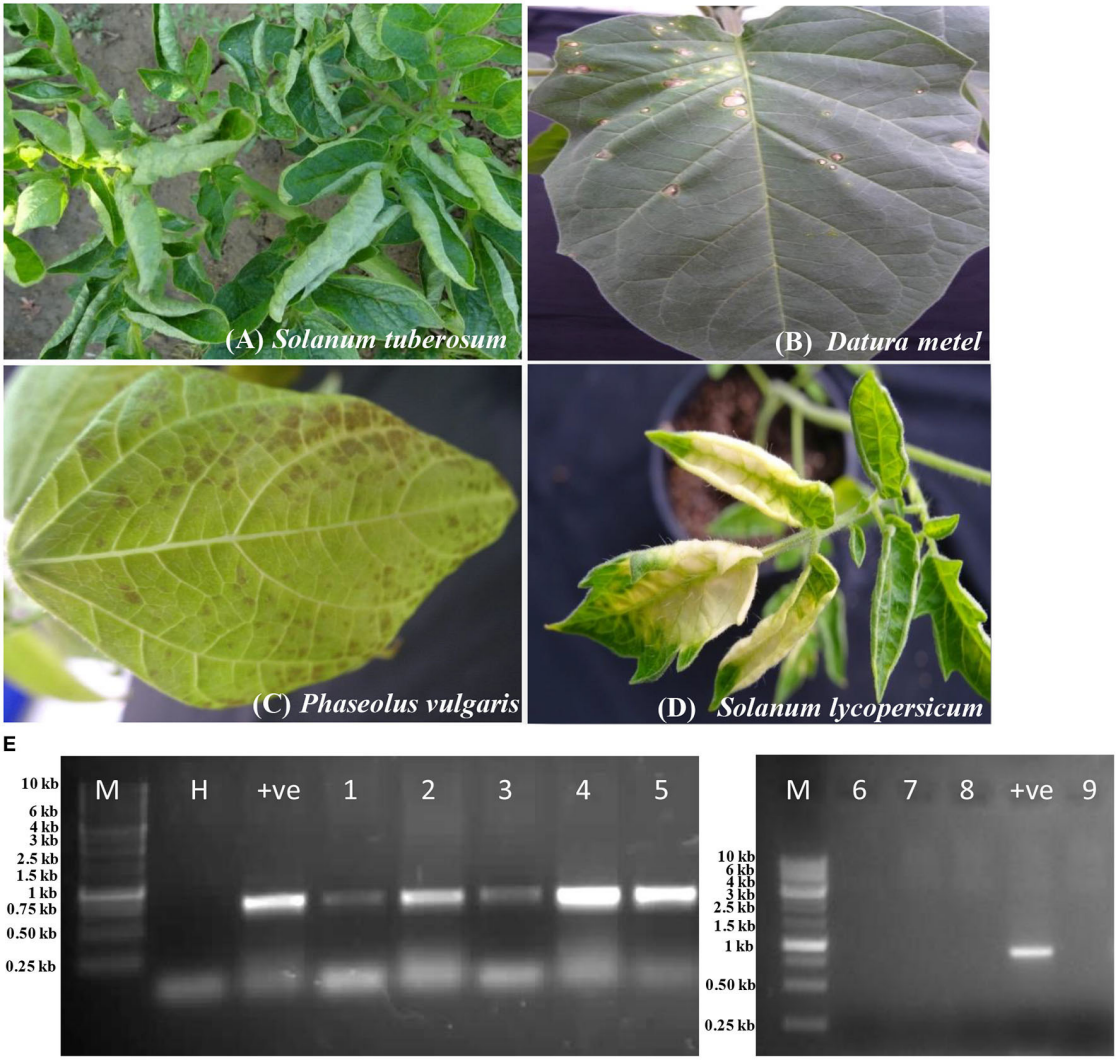
Host reactions of an isolate of *Potato virus* M (PVM-Del-144) upon sap inoculation to various plant species. **(A)** Potato plant showing leaf rolling symptoms in the field. **(B)** *Datura stramonium* showing local lesions. **(C)** *Phaseolus vulgaris* showing necrotic spots. **(D)** *Solanum lycopersicum* showing yellowing of leaves. **(E)** RT-PCR confirmation of virus transmission in inoculated hosts, Lane 1: *Solanum tuberosum*; Lane 2: *Phaseolus vulgaris*; Lane 3: *Datura stramonium;* Lane 4: *Nicotiana benthamiana;* Lane 5: *Solanum lycopersicum*; Lane 6: *Gomphrena globosa;* Lane 7: *Chenopodium quinoa*; Lane 8: *N. glutinosa;* Lane 9: *N. tabacum*. M, 3kb ladder; H, healthy control; +ve, positive control.

The host reactions of PVM-Del-144 following the sap inoculation to the various plant species are presented in Figure 4 and compared with the host reactions of PVM isolates reported from other countries in Table 3. The PVM-Del-144 causes abaxial leaf rolling, mottling of newly developed leaves, and stunting of shoots on potato plants (Figure 4A), whereas PVM isolates reported from other countries develop mottle, mosaic, crinkling, rolling of leaves, and stunting symptoms (Figure 4A, Table 3). The virus isolate was successfully sap-transmitted to four plant species, namely, *Phaseolus vulgaris* cv. Red kidney bean*, Datura stramonium, Solanum lycopersicum*, and *Nicotiana benthamiana*, whereas it was found insusceptible to *Gomphrena globosa, Chenopodium quinoa, N. tabacum* cv. Xanthi, and *N. glutinosa* (Table 3).

Sap inoculation of PVM-Del-144 to *D. stramonium* developed chlorotic lesions on the inoculated leaves (Figure 4B) but did not produce any systemic symptoms, whereas isolates from other countries were reported to cause systemic chlorotic mottling and rugosity (Table 3). All inoculated *P. vulgaris* exhibited faint grayish local lesions at 5–7 days post-inoculation (dpi) that turned to dark brown lesions at 10–12 dpi (Figure 4C) which is similar to the PVM isolates reported earlier from other countries. Similar to PVM isolates reported from other countries, *N. benthamiana* acts as a host for PVM-Del-144 but does not produce any symptoms on inoculated and systemic leaves. However, contrastingly, *S. lycopersicum* developed symptoms of leaf yellowing upon sap inoculation of PVM-Del-144 (Figure 4D), whereas isolates of other countries were reported to be symptomless on this plant (Table 3). All the above host species were positive when examined by RT-PCR (Figure 4E). The comparison of the host reactions of insusceptible plant species for PVM-Del-144 shows that *N. glutinosa* and *N. tabacum* cv. Xanthi are the non-hosts for PVM; however, *G. globosa* and *C. quinoa* were reported to exhibit local chlorotic rings or necrotic spots and chlorotic local lesions, respectively, upon sap inoculation of other PVM isolates (Table 3). All the above mentioned insusceptible plant species to PVM-Del-144 tested negative both in ELISA and RT-PCR (Figure 4E).

## Discussion

Potato is the 3rd major staple food crop in India with 14% of the world's potato production, which ranked 2nd after China (FAOSTAT, 2021). Viruses in addition to the late blight [*Phytophthora infestans* (Mont.) de Bary] are reported to be the most important pathogens affecting potato production worldwide including India (Ahmad et al., 2012). In the current study, DAC-ELISA performed using the commercially available PVM-specific antiserum for the leaf samples collected from the potato field in the Indo-Gangetic plain confirmed the high incidence of PVM up to 70% in the surveyed districts. The present finding supported the previous studies that reported PVM as one of the most prevalent viruses in potato fields in India and worldwide (Jeffries, 1998; Raigond et al., 2013). The overall PVM incidence in the potato fields in India was found to be 18%, which is almost two times higher as compared to the PVM incidence reported in Iran (Tabasinejad et al., 2014).

PVM in India has previously been characterized only on the basis of their biological properties, transmission, and yield loss (Khurana and Singh, 1980); however, till this study, no molecular report for this virus is available from India and a genetic diversity study has not been conducted.

The phylogenetic analyses conducted using the nt sequences of the CP gene of PVM (14 from India and 176 from other countries) together with the most closely related species PVS indicated the diversity among the isolates of PVM and broadly segregated them into three groups (Group-I, Group-II, and Group-III) as compared to the two groups (PVM-o and PVM-d) reported earlier (Tabasinejad et al., 2014); however, the new phylogenetic group (Group-II) contains only two isolates: T20 reported from Slovakia and 20810384 from Hungary. The CP nt sequence identity-based diversity analyses in PVM isolates from India broadly divided them into two categories, in which 10 isolates belong to group I (PVM-o), whereas the remaining four isolates belong to group III (PVM-d). However, further diversity analyses conducted based on the phylogeny showed higher diversity among the isolates from India and were found to be clustered into four different sub-groups. All the four isolates (Del-133, Jau-13, Hat-12, and Bal-21) belonging to group III were clustered in sub-group IIIf, whereas, however, out of 10 isolates of group I, eight (Del-144, Kan-16, Agf-5, Del-147, Del-134, Del-123, Mir-12, and M34) were clustered in sub-group Ic, one (Mat-12) clustered in sub-group If, and a lone isolate (Gaj-13) formed a new sub-group Ig, away from all other isolates reported from India and other countries, indicating higher diversity within the isolates from India. The pairwise comparison of the aa sequences of the CP gene of PVM isolates from India confirms these findings, where 9 out of 10 isolates from PVM-o and four isolates of PVM-d showed similar patterns of aa distribution among their group, respectively, whereas it showed significant differences in aa distribution pattern between the groups. All these analyses indicated that the PVM isolates of India have higher diversity compared to the isolates reported from other countries.

All of the diversity analyses performed in this study clearly indicate that the PVM isolates reported worldwide exhibit significant diversity among themselves. The genetic distances estimated for the entire population and for each phylogenetic group and sub-group indicated that there was significant genetic diversity among the entire population, phylogenetic groups, and sub-groups. The intra- and inter-genetic distance values of the isolates from three phylogroups indicated higher diversity in group III (PVM-d), followed by group II (new) and group I (PVM-o). PVM isolates infecting pepino were found to be stable, whereas significant diversity was recorded in the isolates infecting potato plants. While comparing the diversity within and between populations from individual country/region, isolates from India showed higher diversity within their isolates similar to the isolates from Iran, whereas inter-genetic distance values were highest with the isolates from the United States of America, Bangladesh, and Japan (Tables 5, 6).

The widely used genetic diversity index, *i.e.*, haplotype diversity index (Hd) and nucleotide diversity index (π), was high (Hd ≥ 0.5 and π ≥ 0.005) for all the populations; therefore, based on the description of Grant and Bowen (1998) and considering the value of Hd and π, all the PVM populations are considered to be developed from the same population after a long period of expansion. The intermediate values of the determinants for population differentiation (K_ST_ = 0.172 and F_ST_ = 0.490), calculated for the population divided based on the country of their origin, measure the relative proportions of total genetic diversity attributable to among populations, indicating high genetic differentiation among populations (Balloux and Moulin, 2002). Similarly, the S_nn_ value in this study was found to be maximum (1.000) for the population divided based on the phylogeny, indicating that all three PVM populations in this study are highly differentiated (Hudson, 2000); however, the values of S_nn_ < 1.000 for the populations divided based on the host they infect (0.989) and country of their isolation (0.936) indicate comparatively less diversity between these populations. The Nm value, which is a determinant in studying the gene flow between populations (Whitlock and Mccauley, 1999), was low (0.2–0.26) for the population divided based on phylogeny and country of their isolation, indicating that the gene flow between populations is infrequent and genetic differentiation is large, whereas it was high (0.62) for the population divided based on the host plants they infect, indicating that the gene flow between these two population is frequent, which may be the reason for adapting a new host for PVM.

The selection pressure analyses conducted in this study for the PVM isolates from India showed that the majority of the codons in the CP sequences are under the status of negative (purifying) selection, suggesting that most of the codon mutations in the CP sequence are detrimental and are thus easily eliminated by natural selection (Nielsen, 1999). However, a considerable number of codons (7 by MEME) have also been identified under positive selection which might be the reason for adapting to a new host and environment by this virus (Nielsen, 1999). The neutrality tests performed for PVM populations give mixed results, indicating higher divergence in PVM populations. According to Tajima (1989) and Fu and Li (1993), if the values of the neutrality tests (Tajima's *D*, Fu and Li's *D* and Fu and Li's *F*) are zero or close to zero, it is considered neutral. If these values go higher, it is considered to be a bottleneck or balance selection, but if the values go significantly lower than zero, it is considered a directional selection. In this study, the values of these tests were significantly negative, zero, and significantly positive for the PVM population of phylogroups I, II, and III, respectively, indicating that two major PVM populations deviated from the neutral evolution, had experienced population expansion events in the past, and may still be unstable. A similar observation recorded for the populations divided based on the hosts and country of their origin confirms the above findings (Table 9), of which the PVM population from India showed moderately positive values for these tests, indicating that PVM populations from India deviated from the neutral evolution, experienced population expansion, and have high probabilities to experience more population expansion pressure in future.

The analyses of the complete genome sequence of a lone isolate of PVM (Del-144) from India revealed that the present isolate has a typical genome organization as the other members of the genus *Carlavirus;* however, the genome of Del-144 was found to be 1–62 nt longer than the majority of PVM isolates reported worldwide. Similar to the comparison of CP sequences, the comparison of the complete genome sequence of Del-144 with the sequences of 26 isolates reported worldwide confirms the existence of the same three groups; however, the new phylogenetic group (Group-II) here contains a lone isolate (T20) reported from Slovakia. The study revealed that our isolate clustered together with the isolates of Group-I which is similar to PVM-o; however, within the cluster, it formed a separate branch away from all other isolates, indicating the diversity of the strain.

As the potato grows at low temperatures, the host reaction of the present isolate was examined at a temperature range from 20 to 25°C. The differential host reaction of PVM-Del-144 on the plants viz, *D. stramonium, S. lycopersicum, G. globosa*, and *C. quinoa* compared to the host reactions of PVM isolates reported earlier indicated that the present isolate is different from all other previously reported isolates (Table 3), and these might be used as assay host for future studies. According to the host reaction study performed in this study, *D. stramonium* and *S. lycopersicum* have been identified as local lesion hosts for Del-144 compared to local systemic and symptomless hosts, respectively, for other PVM isolates. The other two plant species, namely, *G. globosa* and *C. quinoa*, were found to be non-host for the isolate Del-144, whereas the same was reported to be a local lesion host for PVM isolates reported earlier (Hiruki, 1970; Xu et al., 2010). All these findings of host reactions confirm the identity of PVM-Del-144 as the divergent strain of PVM occurring in India.

## Conclusion

PVM is widely distributed in the potato fields in India causing yield losses. A number of diverse strains are available in the country that may have differential effects on potato yields, and in future, more diverse stains may emerge that could be a threat to potato production in India. Therefore, strain-specific efficient detection tools and proper management practices should be adopted to manage the virus and associated losses. The complete genome sequence of a new PVM strain (PVM-Del-144) has also been obtained in this study and will be helpful in developing the strain-specific serological and molecular detection tools for timely, routine, and efficient diagnosis of PVM in fields and to produce virus-free potato plants in India, which may help in reducing the yield losses of potato in the country. This study certainly is helpful for understanding the diversity of PVM in India; however, sequencing more number of isolates and performing host reactions of divergent strains followed by developing strain-specific diagnostic tools for managing the disease are recommended for the future.

## Data availability statement

The datasets presented in this study can be found in online repositories. The names of the repository/repositories and accession number(s) can be found in the article/Supplementary material.

## Author contributions

AKu: Conceptualization, Data curation, Formal analysis, Investigation, Methodology, Software, Validation, Writing—original draft, Writing—review & editing. AKa: Conceptualization, Data curation, Investigation, Methodology, Software, Writing—original draft. AJ: Conceptualization, Formal analysis, Methodology, Writing—original draft. AC: Conceptualization, Supervision, Writing—review & editing. BM: Conceptualization, Funding acquisition, Investigation, Project administration, Resources, Supervision, Writing—review & editing.
