# Supplementary material for: Genetic diversity of Potato virus M (PVM) in the major potato growing region in the Indo-Gangetic plain and characterization of a distinct strain of PVM occurring in India
Source: Front Microbiol. 2023 Nov 23;14:1265653. doi: 10.3389/fmicb.2023.1265653 (PMC10701592; doi:10.3389/fmicb.2023.1265653)
Supplement: Supplementary file 1 [file Data_Sheet_1.DOC]

**Supplementary** **Fig 1.** Schematic diagram of the genome organization of PVM together with the details of the primers, and their locations on the genome that have been used to amplify the whole genome of PVM-Del-144 in five overlapping fragments (A), Gel pics showing amplification and cloning of PVM genome in five fragments (B).

**Supplementary** **Fig 2.** Graphical representation of sequence identity matrix of *Potato virus* M based on coat protein nucleotide sequences. Identity scores of coat protein gene are represented in colour-coded blocks prepared by SDT v.1.2 software.
